# Supplementary material for: Deciphering Seasonal Patterns in Animal Feeding: A Mechanistic Approach to Analyzing the Restricted Growth of Iberian Pigs
Source: Animals (Basel). 2024 Nov 27;14(23):3431. doi: 10.3390/ani14233431 (PMC11640408; doi:10.3390/ani14233431)
Supplement: Supplementary file 1 [file animals-14-03431-s001.zip › animals-3279212-supplementary.pdf]

Table S1. Use of the R software environment to fit Gompertz models.

The Gompertz model [4]

$$y(t) = Ae^{-e^{b-kt}}$$

1. To linealize the curve, we fixed the asymptotic weight (*A=228*)

$$\phi = \ln(-\ln(\frac{228}{\text{observed weight}}))$$

   To fit the lineal model we use *lm.*

   model<-lm(ϕ~age, data= data.frame(age, ϕ)

   To obtain the expected weight:

$$Expected\ weight = 228e^{-e^{b-kt}}$$

2. To fit the non-lineal Gompertz model we used *nls*.

   gompertz_model <- nls (observed weight ~ *A* * exp(-exp(*b* - *k* * age)), start = list(*A* = 230, *b* = 1.5, *k* = 0.2), # Approximate initial values; data = data.frame(age, observed weight))
